# Widespread positive but weak assortative mating by diet within stickleback populations

**DOI:** 10.1002/ece3.1609

**Published:** 2015-07-22

**Authors:** Travis Ingram, Yuexin Jiang, Racine Rangel, Daniel I Bolnick

**Affiliations:** 1 Department of Integrative Biology, University of Texas at AustinOne University Station C0990, Austin, Texas, 78712; 2 Howard Hughes Medical Institute, University of Texas at AustinOne University Station C0990, Austin, Texas, 78712

**Keywords:** *Gasterosteus aculeatus*, habitat cosegregation, magic trait, nonrandom mating, sympatric speciation

## Abstract

Assortative mating – correlation between male and female traits – is common within populations and has the potential to promote genetic diversity and in some cases speciation. Despite its importance, few studies have sought to explain variation in the extent of assortativeness across populations. Here, we measure assortative mating based on an ecologically important trait, diet as inferred from stable isotopes, in 16 unmanipulated lake populations of three-spine stickleback. As predicted, we find a tendency toward positive assortment on the littoral–pelagic axis, although the magnitude is consistently weak. These populations vary relatively little in the strength of assortativeness, and what variation occurs is not explained by hypothesized drivers including habitat cosegregation, the potential for disruptive selection, costs to choosiness, and the strength of the relationship between diet and body size. Our results support recent findings that most assortative mating is positive, while suggesting that new approaches may be required to identify the environmental variables that drive the evolution of nonrandom mating within populations.

## Introduction

Assortative mating is a form of nonrandom mating that occurs when male–female mated pairs are more similar (positive assortative mating) or more dissimilar (negative assortative mating) to one another than expected by chance (Cézilly 2004). Positive assortative mating often occurs between species or differentiated populations, reducing hybridization rates and contributing to the maintenance of reproductive isolation (McKinnon et al. 2004; Vines and Schluter 2006; Puebla et al. 2007). Assortative mating can also occur within single undifferentiated populations, where correlations between the traits of male–female pairs have been detected in a wide range of organisms (McLain 1982; Olson et al. 1986; Arnqvist et al. 1996; Roulin 2004; Snowberg and Bolnick 2008). A recent meta-analysis showed that positive assortative mating is common in natural animal populations, with a positive average correlation between male and female traits (*r* = 0.28) and a tendency toward positive assortativeness across taxa for trait categories including size, morphology, and visual signals (Jiang et al. 2013).

Within-population positive assortative mating can have important population genetic consequences. Mating between individuals with similar heritable traits reduces heterozygosity and can increase linkage disequilibrium, inflate quantitative genetic variation, and alter additive genetic covariation among traits (Lynch and Walsh 1998). Positive assortative mating may also contribute to the evolution of reproductive isolation and is a key component of models of sympatric or parapatric speciation with gene flow (Kondrashov and Shpak 1998; Dieckmann and Doebeli 1999; Kirkpatrick and Ravigné 2002). Speciation may occur if traits under divergent selection become linked to traits underlying positive assortment, and should be easiest when positive assortative mating and divergent selection act on the same “magic trait” (Gavrilets 2004; Thibert-Plante and Gavrilets 2013). Contrary to early expectations, magic traits appear reasonably common in nature (Servedio et al. 2011) and can include coloration (Puebla et al. 2007), body size (Conte and Schluter 2013), and host plant (Bush 1969). Thus, assortative mating involving ecologically relevant traits can enhance intraspecific diversity and may play a key role in initiating divergence.

Despite the incidence and importance of positive assortative mating, relatively little work has sought to identify the behavioral, ecological, and evolutionary factors that drive variation in the level of assortativeness among populations (but see McLain 1982; Arnqvist et al. 1996). Assortative mating may be adaptive if individuals gain direct benefits from choosing similar mates – such as reduced risk of injury or sexual cannibalism during mating (Prenter et al. 2006) – or indirect benefits via enhanced offspring fitness. Indirect benefits may occur if disruptive selection reduces the fitness of offspring with intermediate traits, which may drive the evolution of increased assortativeness (Dieckmann and Doebeli 1999; Kirkpatrick and Ravigné 2002; Otto et al. 2008). Assortative mate choice may be an active process in which individuals identify mates similar to themselves, or may occur automatically via spatial or temporal cosegregation if individuals with different traits reproduce in different habitats or at different times (Arnqvist et al. 1996; Snowberg and Bolnick 2012). Assortment can also result from directional sexual selection favoring traits related to fitness or social dominance such as large body size. For example, if large males and large females mate following mutual mate choice or intrasexual competition (Taborsky et al. 2009), small individuals may be left to pair with one another. Where mate preferences exist, assortativeness may be limited by costs to choosiness if high mortality, low population density, or skewed sex ratio increase the risk of failure to mate (Crowley et al. 1991; Tinghitella et al. 2013).

If a factor such as the strength of disruptive selection or the cost to choosiness influences the evolution of assortative mating, we can predict that variation in this factor among populations will result in among-population variation in the strength of assortative mating. Studies measuring assortative mating across a range of conspecific populations (Arnqvist et al. 1996) thus allow tests both of the occurrence of assortative mating in multiple populations and of correlations between assortativeness and hypothesized causal variables. Here, we employ this approach to investigate assortative mating on the basis of a putative magic trait – diet – in 16 lake populations of three-spined stickleback (*Gasterosteus aculeatus*). Using stable isotopes of males and eggs as proxies for male and female diet, we test for assortative mating based on carbon source (littoral vs. pelagic diet) and relative trophic position. We find an average tendency toward positive assortative mating on the littoral–pelagic axis, but magnitudes are weak and not predictable from measured lake properties.

### Study system

Three-spined stickleback have figured prominently in investigations of the role of assortative mating in speciation. Body size likely functions as a magic trait in stickleback speciation, as co-occurring ecotypes (benthic and limnetic in lakes, or freshwater resident and anadromous in streams) are divergent in size, and females prefer size-matched mates (Nagel and Schluter 1998; McKinnon et al. 2004; Conte and Schluter 2013). Body size is the basis for assortative mating in experimental contexts, but it is challenging to directly observe enough mating events in natural populations to confirm the importance of size assortment in wild, undifferentiated populations. Another candidate for a magic trait in stickleback is diet. Stickleback individuals or ecotypes show consistent morphological variation associated with littoral or pelagic foraging, with the length and number of gill rakers positively correlated with the degree of zooplanktivory (Schluter and McPhail 1992; Matthews et al. 2010). In lake stickleback populations, gill raker morphology is frequently subject to disruptive natural selection where littoral or pelagic specialist phenotypes are favored over intermediate trait values. Disruptive selection is strongest in lakes with equitable amounts of littoral and pelagic habitat (Bolnick and Lau 2008). While gill rakers are internal and thus not visible to prospective mates, assortative mate preference might involve diet itself, if females prefer males similar to themselves in foraging behavior or diet-derived olfactory cues. Stickleback have been found to preferentially associate with mates on the basis of similarity in olfactory cues (Ward et al. 2004; Rafferty and Boughman 2006), and these cues may themselves be mediated by effects of diet on gut microbiota (Bolnick et al. 2014). Alternatively, assortative mating by diet may arise through spatial cosegregation of individuals with similar diets (Snowberg and Bolnick 2012), potentially involving female preference for particular nest microhabitats (Bolnick et al. 2015). Finally, mate choice may be based on visible traits such as body size or shape that are also correlated with diet (Bolnick and Paull 2009; Head et al. 2013).

Two studies have tested for positive assortative mating in stickleback in lakes containing a single stickleback population each, by measuring the correlation between male and female diet using stable isotope analysis (Snowberg and Bolnick 2008, 2012). This is possible in unmanipulated populations because male stickleback guard nests with fertilized eggs, which have stable isotope values that reflect those of the female that deposited them (Grey 2001; Snowberg and Bolnick 2008, 2012) In aquatic systems, stable isotopes of carbon (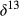
C) indicate the use of littoral versus pelagic prey, while nitrogen isotope values (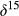
N) indicate relative trophic position (Post 2002). Stickleback within populations consistently show significant individual variation in diet that is correlated with isotopic variation (Snowberg et al. 2015), so collection of males and nests allows estimation of two key dietary niche measures for mated pairs of males and females. The first study using this approach found a positive male–egg isotope correlation (Snowberg and Bolnick 2008), while the second also detected positive assortative mating (*r* = 0.28) and found that habitat cosegregation accounted for some but not all of this correlation (Snowberg and Bolnick 2012). The present study builds upon these results by measuring assortative mating by diet in 16 populations and asking whether assortativeness can be predicted by variables representing the expected strength of disruptive selection or the potential costs of choosiness.

## Methods

### Sampling

We measured assortative mating by diet in 16 lakes in southwestern British Columbia during the stickleback breeding season (May 27–July 8, 2013). Fifteen lakes are on northern Vancouver Island, in several watersheds – Campbell (6 lakes), Amor de Cosmos (5), Mohun (2), Pye (1), and Brown's Bay (1) – and the final lake is on nearby Quadra Island. Lakes were selected to represent a wide range of lake sizes (4.5- to 574-ha surface area) across multiple watersheds (Table 1). Each of these lakes contains a single stickleback population: There is no evidence of discrete genotypic or phenotypic clusters in any lake in these watersheds (Caldera and Bolnick 2008; Snowberg et al. 2015).

**Table 1:** Properties of lakes used in this study

Lake	Watershed	Latitude	Longitude	SA (ha)	P (m)	LP	Pred.	MNND 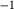	Mass- 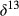	Mass- 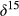 N 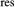
Amor	Amor de Cosmos	50 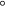 09 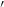 27 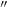 N	125 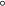 34 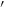 42 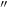 W	329.9	20,130	61.0	1.2	0.17	**0.47**	**0.26**
Blackwater	Amor de Cosmos	50 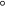 10 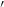 40 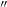 N	125 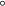 35 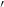 20 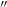 W	37.5	5750	153.3	0.0	0.12	0.16	**0.27**
Boot	Campbell	50 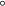 02 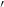 55 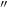 N	125 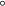 31 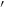 22 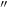 W	98.7	6325	64.1	1.6	0.18	0.05	0.07
Brown's Bay	Brown's Bay	50 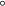 09 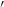 29 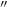 N	125 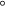 24 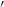 57 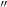 W	7.9	1750	221.5	0.0	0.17	0.04	**0.64**
Cranberry	Mohun	50 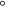 05 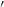 21 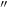 N	125 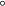 27 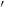 17 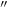 W	5.7	1400	245.6	0.3	0.12	*0.24*	−0.15
Echo	Campbell	49 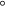 59 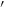 19 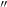 N	125 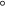 24 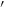 38 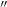 W	20.6	3150	152.9	0.5	0.26	−0.20	0.21
Gosling	Campbell	50 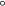 02 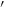 43 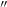 N	125 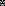 40 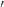 41 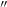 W	62.5	6608	105.7	1.0	0.32	0.12	−0.17
Gray	Campbell	50 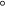 03 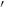 27 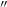 N	125 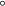 35 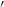 40 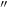 W	52.9	5320	100.6	0.7	0.12	**0.34**	−0.03
Lawson	Campbell	50 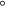 02 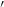 17 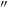 N	125 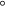 31 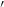 29 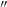 W	22.9	2810	122.7	0.2	0.14	0.08	**0.28**
Little Mud	Amor de Cosmos	50 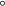 12 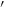 23 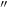 N	125 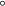 33 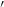 00 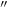 W	4.4	1012	230.0	0.7	0.03	−0.18	0.12
Merrill	Campbell	50 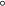 03 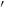 34 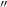 N	125 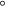 33 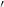 21 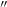 W	65.6	3600	54.9	0.7	0.11	0.10	0.17
Mohun	Mohun	50 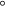 09 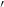 47 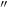 N	125 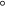 29 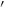 18 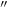 W	620.9	31,207	50.3	2.5	0.18	0.14	*0.22*
Ormond	Amor de Cosmos	50 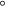 10 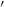 49 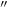 N	125 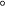 31 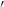 30 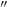 W	5.6	1230	219.6	0.2	0.11	0.05	**0.42**
Pye	Pye	50 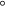 17 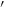 38 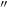 N	125 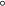 34 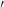 28 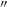 W	369.8	13,200	35.7	1.3	0.09	0.15	**−0.32**
Roberts	Amor de Cosmos	50 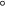 12 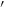 58 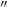 N	125 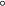 32 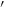 30 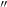 W	160.0	8175	51.1	0.4	0.24	*0.24*	−0.13
Village Bay	Village Bay	50 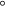 10 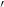 31 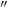 N	125 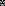 11 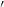 26 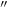 W	76.0	9900	130.3	2.3	0.24	−0.07	**0.32**

LP is the lake perimeter:area ratio. Pred. is the average density of potential predators (trout and sculpin) per 150 m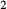 snorkel transect, and MNND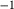 is a measure of the density of nests in the nesting habitat. Mass-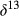 and Mass-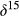
N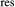 are the correlation coefficients between each isotope and male body mass, with italics indicating marginally significant correlations (0.05 < *P* < 0.10) and boldface indicating significant correlations (*P* < 0.05) within lakes.

In each lake, snorkelers identified nesting habitat in the littoral zone and searched for males guarding nests. Males were only collected if their nests were found to contain eggs. The spatial location of each nest in UTM was determined using a Garmin eTrex 10 handheld GPS device (Garmin Ltd., Olathe, KS) accurate to ∼3 m. The nest depth was measured to 5 cm, and the presence of emergent macrophytes such as lilies or submerged macrophytes such as stonewort (*Chara* sp.) within 1 m of the nest was recorded. The male was euthanized with an overdose of MS-222 and weighed to 0.01 g with an electronic balance. A small piece of male dorsal muscle tissue and several eggs from each clutch were placed in dry microcentrifuge tubes. These samples were stored on ice in a cooler for no more than 8 h and then dried at 60
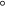
C for 48 h in preparation for stable isotope analysis.

To confirm that egg isotope values are a suitable proxy for female diet (see also Grey 2001; Snowberg and Bolnick 2008, 2012), we sampled gravid females from three lakes in which they were sufficiently abundant (Blackwater, Little Mud, and Roberts). Gravid females were collected with minnow traps and dip nets and euthanized with MS-222, and then, 5–10 eggs were extruded. Eggs and muscle clips from females were stored and dried as for males.

### Stable isotope analysis

For each dried muscle or egg tissue sample, we packaged a ∼1 mg subsample into a tin capsule for stable isotope analysis. 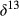
C and 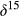
N were measured at the University of California Davis stable isotope facility using a PDZ Europa ANCA-GSL elemental analyzer interfaced to a PDZ Europa 20-20 isotope ratio mass spectrometer (Sercon Ltd., Cheshire, UK). Isotope ratios are expressed in the conventional “delta” notation as the differences in heavy:light isotope ratios relative to standards (Pee Dee Belemnite carbon and atmospheric nitrogen). If multiple clutches of eggs were present in a nest, we averaged isotope values for subsequent analysis. Precision, measured as the standard deviation of internal replicates during each batch, ranged from ±0−0.20‰ for 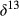
C and ±0.04−0.29‰ for 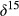
N.

We measured the position of stickleback on the littoral–pelagic axis using 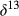
C values. We then used a separate linear regression of 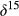
N against 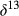
C for each of males and eggs in each lake to obtain residual 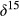
N (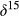
N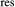) as a measure of relative trophic position. By removing any relationship with 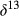
C, we account for variation among lakes in the degree to which the isotopes covary. Assuming the change in 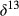
C with trophic transfer is near zero (Post 2002), 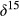
N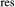 captures trophic level variation independent of variation in carbon source. It is common to convert these values to more ecologically intuitive variables, proportion of littoral carbon and trophic position, by comparing them to baseline isotope values from primary consumers such as mussels and snails (Post 2002). However, the scarcity or absence of mussels or snails in some lakes meant these values could not consistently be calculated. We also explored the use of principal component analysis (PCA) to identify the major axis of isotopic variability within each lake, similar to Snowberg and Bolnick (2008). We centered 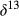
C and 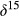
N around zero separately for males and for eggs in each lake, to remove any differences between male and egg means. Then, for each lake we ran a PCA on the covariance matrix of centered 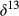
C and 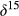
N and used PC1 to quantify the major axis of isotopic variability.

### Measuring assortative mating by diet

The strength of assortment based on a continuous trait is often measured as the Pearson's correlation between male and female trait values across mated pairs (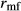). As we use egg isotope values as a proxy for female diet (Arnqvist et al. 1996; Whitlock 2005), we calculated the correlation coefficient (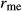) and significance of the male–egg correlation for 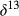
C and 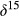
N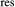 in each lake, and generally refer to 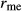 as the strength of assortative mating. We repeated this calculation using isotope PC1 for each lake. These values underestimate 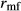 because the female–egg correlation (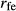) is imperfect, so we also calculated the expected male–female correlation as 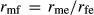 (Snowberg and Bolnick 2008) using the average of the three estimates of 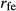 obtained with a *z*-transformation (Corey et al. 1998).

To test whether assortment tended to be positive, we used a weighted *Z*-test to combine *P*-values from the within-lake correlation tests (Arnqvist et al. 1996; Whitlock 2005), and a *t*-test to determine whether the average of the 16 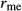 estimates for either isotope differed from zero. We first tested for a watershed effect by asking whether 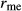 differed between the two watersheds represented by more than two lakes: Amor de Cosmos (*n* = 5 lakes) and Campbell (*n* = 6); as we found no such effect, we treated each lake as independent for the purpose of these analyses. We also compared the mean and variance of the observed 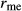 values to null values obtained by simulation. We sampled 16 sets of random bivariate normal deviates with the sample sizes from each lake, calculated means and variances of 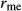, and repeated this 9999 times to obtain null distributions. We first generated data with an underlying correlation of zero in each population to ask whether the observed mean 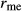 was greater than expected by chance. We then generated data where each underlying correlation matched the observed mean 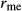 for 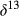
C or 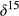
N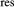, and asked whether the variance in 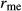 exceeded the null expectation if all lakes had the same 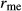. All statistical analyses were carried out in the R environment (R Core Team 2014).

### Partitioning effects of habitat cosegregation

We tested for associations between habitat variables and isotope values to assess whether any positive assortative mating could result from similar males and females using the same microhabitats and mating due to spatial proximity. We focused on two habitat characteristics that often differ between nests of benthic and limnetic stickleback populations, water depth, and proximity to vegetation (benthic stickleback tend to nest in deeper water and closer to cover; Ridgway and McPhail 1984; Vines and Schluter 2006). We categorized nests as being near living vegetation if they were within 1 m of emergent or submerged macrophytes, with nests not near vegetation typically associated with bare substrate or sunken logs.

We fit separate linear models for male 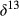
C, egg 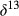
C, male 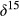
N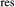, and egg 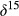
N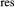 in each lake, with depth (continuous) and vegetation (binary) as predictors. We took residuals from each model to statistically remove any isotopic variation explained by habitat characteristics (Snowberg and Bolnick 2012). We recalculated the correlation between residual male 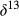
C and residual egg 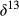
C, and between residual male 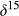
N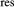 and residual egg 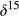
N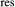 in each lake, and repeated the weighted *Z*-tests and *t*-tests for overall tendencies for the correlation to differ from zero.

### Potential predictors of assortativeness

If assortative mating evolves as an adaptive response to indirect selection on mate preference, disruptive selection should lead to greater assortativeness (Dieckmann and Doebeli 1999; Kirkpatrick and Ravigné 2002). Disruptive selection on stickleback trophic morphology is stronger in lakes with an intermediate littoral–pelagic ratio (LP), calculated as the perimeter divided by the surface area (Bolnick and Lau 2008). LP is larger in smaller lakes and in lakes with more reticulated shorelines. We used the perimeter and surface area of each lake from bathymetry maps or satellite imagery to calculate LP in m/ha. We then fit linear models to test whether the strength of assortative mating (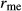) for either isotope was predicted by LP or 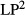. We predicted that if disruptive selection drives positive assortative mating, we should see a negative quadratic term, indicating that assortative mating is more prevalent in lakes with more equitable amounts of littoral and pelagic habitat (intermediate LP).

Costs of assortative mating are likely when high mortality risk or low density of potential mates result in an elevated risk of failure to mate (Crowley et al. 1991). We used visual snorkel transects to estimate the density of other fish species – cutthroat trout (*Oncorhyncus mykiss*) and prickly sculpin (*Cottus asper*) – that are known predators of stickleback (Reimchen 1994). Between 3 and 8 snorkelers swam a 75-m transect through the nesting habitat in each lake at haphazardly selected times. Each snorkeler swam in a straight line at a steady pace, counting all trout and sculpin visible ahead of or below them within 1 m on either side of the transect line. We calculated the average density of trout and sculpin observed per transect for each lake. We also estimated the density of nesting males available for females to evaluate using the GPS coordinates of each nest. We calculated the distance in meters between each nest and its nearest neighbor and used the inverse of the mean nearest neighbor distance (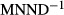) as a measure of nesting male density. We fit separate linear regressions for to ask whether either measure of the potential cost to choosiness was related to assortative mating for either isotope, predicting that high predatory fish density and low nest density would decrease assortativeness.

To assess whether male–egg isotope correlations occur because isotopes are related to other traits, we measured the strength of the relationship between each isotope and body mass in each lake. We focused on body mass of males, which is associated with both diet and mating preference in many stickleback populations, predicting that if assortative mating was driven by body size, lakes with tighter size–diet relationships should show stronger assortative mating. We measured the correlation between male mass and each of male 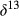
C or 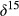
N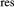 in each lake, and used a linear regression for each isotope to test whether assortative mating was stronger in lakes with a stronger male mass–isotope correlation for that isotope. While we could not measure female mass-isotope correlations for all populations, we did measure these values for the three lakes with female samples.

## Results

### Measuring assortative mating by diet

**Figure 1:**
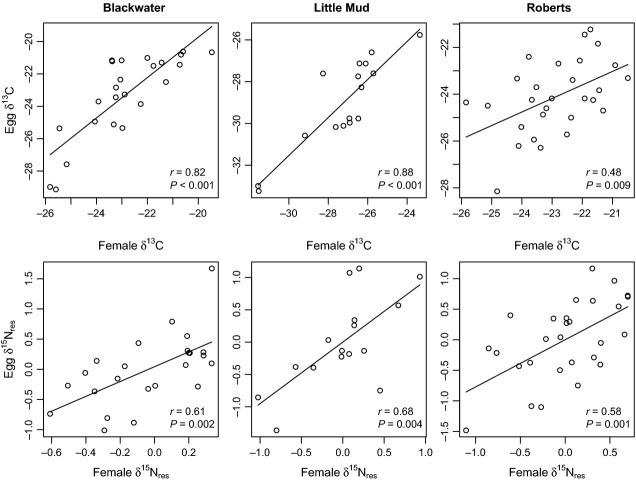
Relationships between female and egg 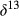
C and 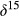
N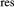 in three lakes. Best-fit regression lines are shown for visual effect.

Female–egg isotope correlations were strong in the three lakes in which we collected gravid females, confirming that egg isotopes can serve as a proxy for female diet (Fig. 1). For 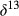
C, we found significant female–egg correlations in Blackwater Lake (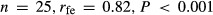), Little Mud Lake (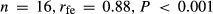), and Roberts Lake (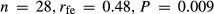). The average 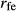 was estimated to be 0.77 using Fisher's *z*-transform, and pairwise comparisons showed that the correlation in Roberts Lake was lower than that in Blackwater (*P* = 0.04) or Little Mud Lakes (*P* = 0.01). For 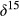
N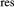, the three estimates were 0.61, 0.68, and 0.58 (all *P* < 0.004), with an average 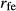 of 0.62 and no difference between the three lakes.

**Figure 2:**
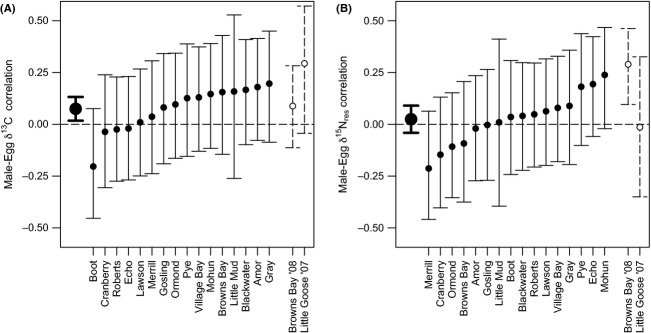
Assortative mating based on 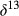
C (A) and 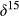
N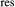 (B). Estimates (filled symbols) and 95% confidence intervals for the male–egg correlation are shown for correlations within each lake, with lakes ordered by the value of the correlation coefficient. The result of a one-sample *t*-test across lakes (mean and 95% confidence interval) is shown at the left of each panel by large symbols and thick lines. For comparison, values recalculated from two previous studies in this area are indicated to the right of each panel by open symbols and dashed lines..

We collected between 52 and 62 male–egg pairs from all lakes except for Little Mud Lake, which only yielded 24 pairs in the time available. Within lakes, correlations between male and egg isotope values were weak for both 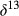
C (Figure S1) and 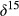
N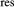 (Figure S2), with 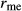 ranging from −0.20 to 0.20 for 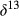
C and from −0.24 to 0.23 for 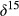
N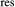 (Table 2). Converted to expected male–female correlations, 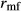 ranged from −0.27 to 0.26 for 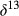
C and from −0.37 to 0.35 for 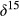
N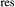. Male–egg correlations were not significantly different from zero in any individual population (all *P* > 0.15 for 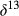
C and all *P* > 0.06 for 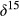
N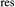; Fig. 2). Measures of assortative mating estimated using 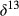
C and 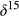
N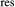 were not correlated across lakes (*r* = 0.14,*P* = 0.60). 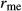 did not differ significantly between the Amor de Cosmos and Campbell watersheds for 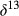
C (two sample *t*-test: *t* = −1.5, *P* = 0.17) or 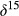
N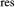 (*t* = 0.54, *P* = 0.61).

**Table 2:** Correlations between male and egg isotopes (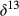
C and 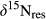) in each lake, both before and after adjustment to remove statistical associations with habitat variables (nest depth and presence of vegetation)

		Raw isotope values				Habitat-adjusted			
		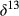 C		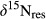		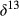 C		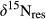	
Lake	N	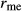	P	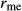	P	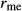	P	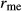	P
Amor	60	0.18	0.17	−0.02	0.88	0.21	0.11	−0.03	0.81
Blackwater	58	0.17	0.22	0.04	0.76	0.17	0.22	0.06	0.67
Boot	52	−0.20	0.15	0.04	0.81	−0.21	0.14	0.04	0.79
Brown's Bay	52	0.16	0.31	−0.09	0.55	0.17	0.26	−0.05	0.74
Cranberry	52	−0.04	0.80	−0.15	0.30	−0.05	0.75	−0.15	0.26
Echo	62	−0.02	0.88	0.19	0.13	−0.02	0.87	**0.26**	**0.04**
Gosling	56	0.08	0.56	0.00	0.98	0.07	0.63	0.00	1.00
Gray	58	0.20	0.17	0.09	0.54	0.22	0.12	0.06	0.68
Lawson	58	0.01	0.94	0.06	0.64	0.02	0.91	0.07	0.61
Little Mud	24	0.16	0.46	0.01	0.96	0.16	0.46	−0.06	0.77
Merrill	53	0.04	0.80	−0.21	0.13	0.02	0.86	−0.22	0.12
Mohun	61	0.15	0.27	*0.24*	*0.07*	0.11	0.42	*0.24*	*0.07*
Ormond	61	0.10	0.47	−0.11	0.42	0.11	0.43	−0.14	0.30
Pye	51	0.13	0.38	0.18	0.21	0.10	0.50	0.17	0.23
Roberts	62	−0.03	0.85	0.05	0.71	−0.02	0.86	−0.01	0.94
Village Bay	61	0.13	0.33	0.08	0.55	0.17	0.20	0.03	0.84

Italics highlight marginally significant correlations (0.05 < *P* < 0.10) and boldface highlights significant correlations (*P* < 0.05).

Across all lakes, the mean 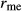 for 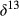
C was significantly greater than zero (mean 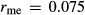; weighted *Z*-test: *Z* = 2.03,*P* = 0.043; *t*-test: 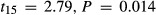; Fig. 2). There was no tendency for the male–egg correlation for 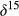
N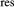 to differ from zero (mean 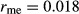; weighted *Z*-test: *Z* = 0.90,*P* = 0.37; *t*-test: 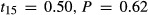). Correlations calculated using isotope PC1 very closely mirrored the results for 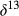
C, although the weighted *Z*-test became marginally nonsignificant (mean 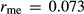; weighted *Z*-test: *Z* = 1.94, *P* = 0.052; *t*-test: 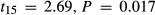). These conclusions are supported by the simulations assuming a true 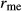 of zero: The observed mean 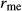 was greater than expected for 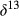
C (*P* = 0.037), but not for 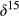
N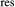 (*P* = 0.46). When we simulated data for each lake using the observed overall mean 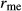, we found that the observed variance among lakes in 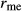 did not differ from the null expectation for either 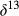
C (*P* = 0.26) or 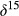
N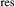 (*P* = 0.61).

### Partitioning effects of habitat cosegregation

Nest depths ranged from 15 to 250 cm from the surface, except for one very deep nest at ∼4 m. In total, 49% of nests were within 1 m of the nearest submerged or emergent vegetation, with lakes varying from 15 to 92% of nests near vegetation. There were some significant associations between 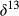
C and 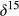
N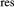 and the two habitat variables (Table A1), but these were generally not consistent among lakes and never explained much variation in 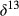
C or 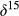
N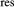 (maximum 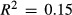).

Accounting for the variation explained by habitat had no tangible effect on our results. One correlation for male and egg 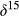
N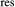 became weakly significant, but the range of variation in 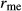 was almost unchanged. The significant tendency for 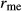 to be positive for 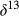
C remained (mean 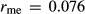; weighted *Z*-test: *Z* = 2.08, *P* = 0.037; *t*-test: 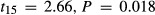), while the average correlation for 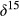
N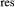 remained close to zero (mean 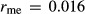; weighted *Z*-test: *Z* = 0.74, *P* = 0.46; *t*-test: 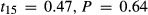)

### Potential predictors of assortativeness

Lakes varied in littoral–pelagic ratio LP (36−246 m/ha), density of potential predators (0−3.7 fish per transect), nest density (0.031 < 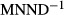 < 0.32 m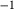), and the correlation between male mass and isotope values (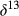
C: −0.20 < *r* < 0.47; 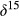
N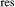: −0.32 < *r* < 0.64). Where significant relationships between mass and 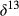
C or 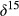
N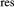 occurred, they were usually positive (*P* < 0.05 for 2 of 16 lakes for 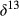
C and for six of 16 lakes for 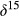
N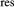, with only one lake showing a significant negative mass–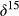
N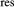 relationship. There were no significant relationships between female mass and isotopes in the three lakes with female data (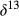
C: −0.099 < *r* < 0.18; 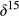
N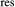: −0.13 < *r* < 0.018; all *P* > 0.3).

**Figure 3:**
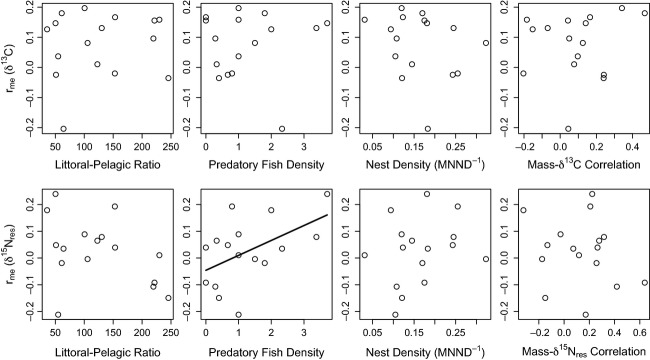
Scatterplots relating the strength of assortment (male–egg correlation for 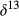
C or 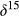
N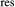 to four potential predictors: lake littoral–pelagic ratio, predatory fish density, nest density, and the strength of the male size–isotope relationship. The only significant predictor was the predatory fish density (for 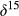
N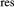).

The potential predictors were not significantly correlated with one another (−0.3 < *r* < 0.4, *P* > 0.1), except for LP and predator density, which were negatively correlated (*r* = −0.55, *P* = 0.029). None of the variables – littoral–pelagic ratio (LP and 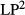), predator density, nest density, or strength of mass–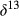
C relationship – showed associations with the strength of assortative mating (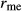) for 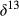
C (Fig. 3; all *P* > 0.30). For 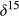
N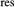, there was a weakly significant relationship between 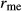 and predator density, implying an increase in 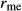 of 0.056 for each additional predator per 150 m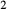 (*P* = 0.044, 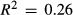; Fig. 3). However, this effect was opposite the predicted direction, and other regressions were nonsignificant.

## Discussion

Our survey of 16 stickleback populations in postglacial lakes found an overall tendency toward positive assortative mating based on carbon stable isotopes, a measure of littoral versus pelagic diet. However, within each lake, the male–egg isotope correlations were weak relative to the 93% of published studies that have reported significant within-population assortative mating (average 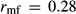; Jiang et al. 2013). The strength of assortative mating was not explained by our hypothesized drivers, but this is likely in part because there was little variation among lakes in assortativeness.

Within most lakes, the correlation between male and egg 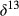
C was either weakly positive or very close to zero, with only one lake (Boot Lake) showing a trend toward a negative correlation. This lake does not appear to differ substantially from the others in factors that might lead to negative assortment. While the range of estimates of 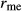 for 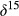
N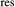 was comparable to that for 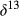
C, values were more evenly distributed around zero and there was no tendency toward positive or negative assortment. The lack of assortment based on 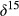
N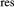 is unsurprising, as our measure of relative trophic position deliberately removes variation associated with the main littoral–pelagic axis of diet variation in stickleback. The lack of a cross-population correlation between 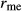 for carbon and nitrogen isotopes also means we see no evidence for more complex assortative mating involving multiple dietary axes.

Unlike two previous studies of single lakes in this region (Snowberg and Bolnick 2008, 2012), we did not detect any significant within-lake assortative mating based on stable isotopes of males and eggs. Some of this may come from differences in analysis. Snowberg and Bolnick (2008) found a significant male–egg correlation on a single isotopic dimension (isotope PC1; 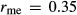). If we reanalyze this data using our approach of examining 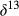
C and 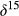
N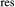 separately, there remains a positive male–egg correlation for 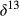
C which is marginally nonsignificant but nonetheless larger than any 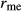 in the present study. Snowberg and Bolnick (2012) examined assortative mating in Browns Bay Lake in terms of trophic position and proportion littoral carbon and accounted more thoroughly for spatial and habitat heterogeneity. While we cannot calculate these niche measures without complete isotopic baseline data, when we reanalyze Snowberg and Bolnick (2012)'s isotope data using our approach, we confirm a significant positive correlation for 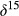
N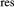 that is also stronger than we observed for any lake in the current study including Brown's Bay Lake. This raises the question of why assortativeness might vary over time, and whether the male–egg correlations might be stronger if the 16 lakes were resampled in other years. The strength of disruptive selection can vary among years in these populations (Bolnick and Lau 2008), and other potential drivers of assortativeness are also likely to vary temporally. Anecdotally, populations in the 2013 season began breeding later than in previous field seasons: If external forcers such as climatic variability have some impact on the extent of assortativeness through effects on breeding time, both longitudinal and cross-sectional surveys may be required to tease apart the drivers of assortativeness.

If weak but real assortative mating was occurring within populations, we likely lacked the sample size needed to rule out random mating. To reliably detect assortative mating (power of 0.8) within a population with the average 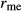 we observed for 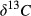, we would have needed over 1000 samples, and even to detect the strongest 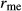 of 0.20 would require over 200 samples. This would be difficult in this system without either adversely impacting stickleback populations or sampling over such an extended period of time that temporal variation in mating behavior and isotope values would complicate interpretation. The meta-analytic approach we used was therefore important in allowing detection of weak assortment. Other factors may have limited our ability to detect any assortative mating that occurred within lakes. Male stickleback are known to steal eggs from other males' nests and place them in their own, meaning eggs are not guaranteed to have been fertilized by the male guarding the nest (Jamieson and Colgan 1992). If many male–egg pairs did not represent cases of mate choice, a signal of assortativeness could be obscured. Noise in the isotopic data – including imperfect correlations between isotopic variation and diet variation (Bolnick and Paull 2009; Snowberg et al. 2015) and nondietary factors such as starvation that can affect isotope values (Bowes et al. 2014) – might also have reduced our ability to detect male–egg isotope correlations.

Habitat cosegregation based on nest depth and the presence or absence of nearby living vegetation explained a small amount of the variation in isotope ratios for both males and eggs in most lakes. Some but not all significant relationships were consistent with predictions based on differences in nesting habitat among benthic-like and limnetic-like populations. Benthic stickleback tend to nest deeper and in association with vegetation (Ridgway and McPhail 1984; Vines and Schluter 2006), suggesting that 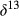
C should increase with both habitat variables, but these relationships were not consistently found in this study (Table S1). We had no a priori predictions for how 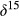
N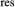 would relate to the habitat variables, and this analysis also yielded scattered relationships. Presumably due to the weak habitat–isotope associations, accounting for the isotopic variation that could be explained by habitat had no qualitative effect on our conclusions, as the relationship between the residual male and egg 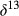
C remained weak within lakes but positive when we combined results across lakes.

Our analyses failed to support hypotheses about factors that would predict the extent of assortative mating, leaving us without a clear explanation for the overall tendency toward positive assortative mating. The only significant predictor variable was predatory fish density, but this relationship was opposite to the hypothesized direction (higher risk of predation associated with greater assortativeness) and was only seen for 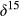
N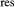, which showed no overall evidence for assortative mating. Our ability to identify any predictors that contributed to the overall positive male–egg correlation for 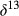
C was likely weakened by low variation in assortativeness among populations, and by the need to use indirect measures of disruptive selection and costs to choosiness. A possible explanation for the positive average male–egg correlations is that some cosegregation or spatial isotopic variability was not captured by the habitat variables we measured. Temporal variability in isotope values is less likely to play a major role given the short timescale over which each lake was sampled (typically only a few days, with a maximum of 2 weeks between the first and last sample collected). If mate preference does drive the tendency toward average assortative mating by diet, it may do so via correlations with other traits. If size-based assortative mating, as documented between stickleback ecotypes (Nagel and Schluter 1998; McKinnon et al. 2004), also occurs in undifferentiated populations, it could leave a signal in the male–egg isotope correlation due to relationships between size and diet. However, while we found significant male size–isotope relationships in several lakes that generally indicate that littoral prey use and trophic position increase with size (see also Matthews et al. 2010), the strength of these associations did not predict the degree of assortative mating measured with isotopes. Size preference may still play a role in assortative mating in our lakes, but is likely to be relatively weak and may occur in concert with mate discrimination based on other factors such as body shape, condition, color, or habitat (Snowberg and Bolnick 2012; Head et al. 2013). Finally, the weak assortative mating we observed on the littoral–pelagic axis may be more directly related to dietary similarity, potentially resulting from assortative schooling or preference for similar diet-derived olfactory cues (Ward et al. 2004; Rafferty and Boughman 2006; Bolnick et al. 2014), but in these populations any such effect appears to be weak.

The low typical magnitude of assortative mating may help to explain why sympatric speciation does not generally occur in solitary lake stickleback populations despite what should be favorable conditions: disruptive selection and positive assortative mating based on a “magic trait” or trait complex. A simple sympatric speciation model tailored to the stickleback system found that speciation was only expected if the male–female trait correlation was at least 0.5 (roughly double our highest estimate; Bolnick 2011), and even then only given stronger disruptive selection than occurs in these populations (Bolnick and Lau 2008). This model did not allow disruptive selection to drive the evolution of increased assortative mating, an important component of similar models. However, if disruptive selection drives the evolution of increased assortativeness, we predicted that populations in lakes more conducive to disruptive selection (with a roughly equitable distribution of littoral and pelagic habitat) would show stronger assortative mating, but this was not observed (see also Jiang et al. 2013).

Our study design included both a meta-analysis of the strength of assortative mating among many replicate populations (see also Arnqvist et al. 1996) and tests of hypothesized drivers of assortativeness. An understanding of these drivers is of great theoretical interest, but will also have practical implications. For example, assortative mating based on origin (wild vs. captive-reared) has the potential to hinder reintroductions of endangered species (Slade et al. 2015), and reduced assortativeness due to habitat modification can threaten the integrity of young species (Seehausen 2006). We did not find support for the hypothesized drivers of assortative mating, likely in part because there was insufficient variation in the assortativeness among populations. This result highlights the importance for future comparative studies of this type to identify systems with sufficient interpopulation variation in the strength of assortative mating in addition to variation in the hypothesized drivers. A combination of robust comparative surveys and experimental manipulations is likely to be the best way forward if we are to understand when the genetic consequences of assortative mating are most likely to occur.

## Supporting Information

**Figure S1**. Relationships between male and egg C in each of 16 lakes.

**Figure S2**. Relationships between male and egg N in each of 16 lakes.

**Table S1**. Results of linear models using habitat characteristics (nest depth and the presence of vegetation) to predict male and egg C and N.
