# Supplementary figures and images for: Widespread positive but weak assortative mating by diet within stickleback populations
Source: Ecol Evol. 2015 Jul 22;5(16):3352–63. doi: 10.1002/ece3.1609 (PMC4569031; doi:10.1002/ece3.1609)

Egg  $\delta^{13}\text{C}$

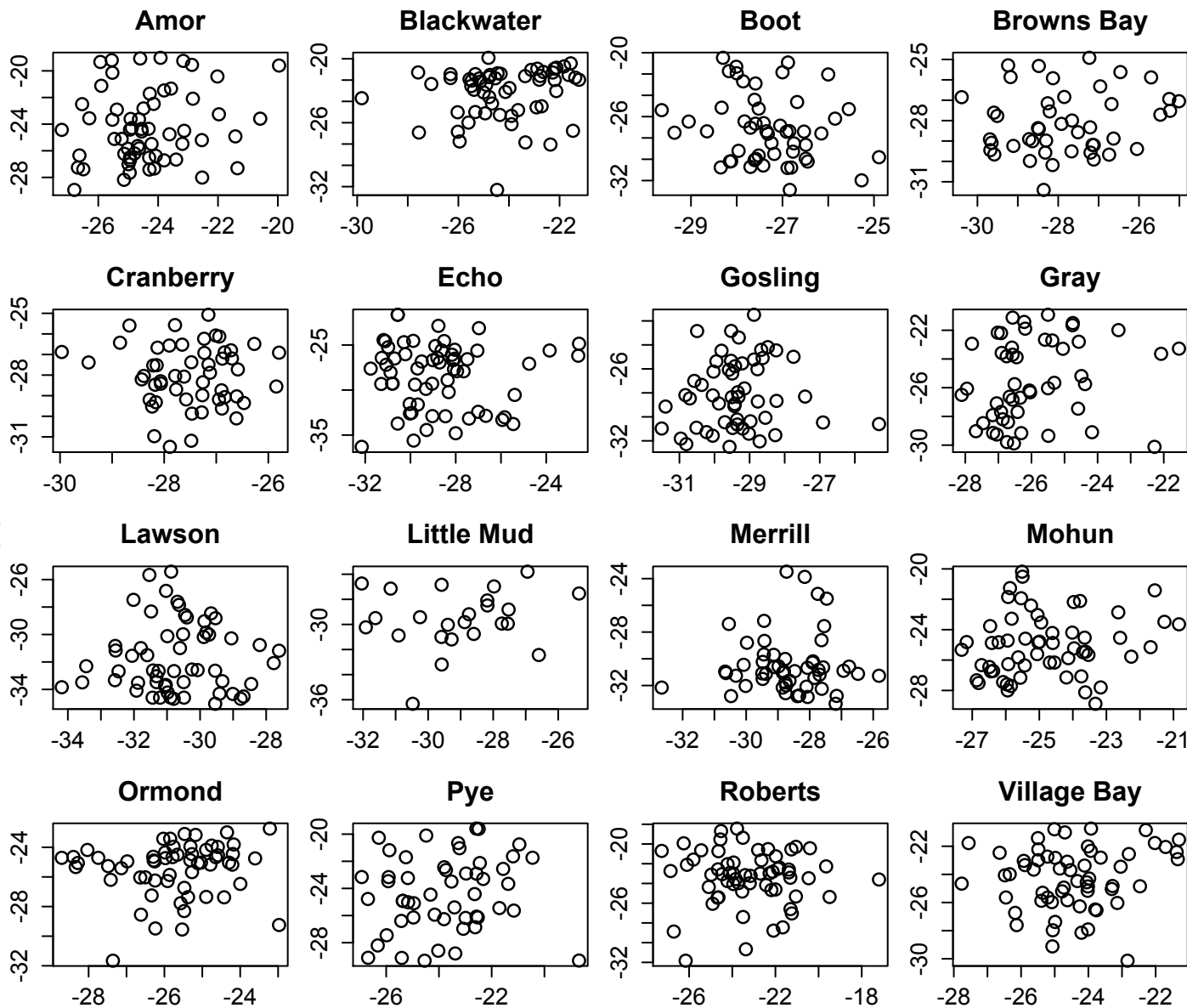

Male  $\delta^{13}\text{C}$

Supplement: Supplementary file 1 — Figure S1. Relationships between male and egg C in each of 16 lakes. [file ece30005-3352-sd1.pdf]

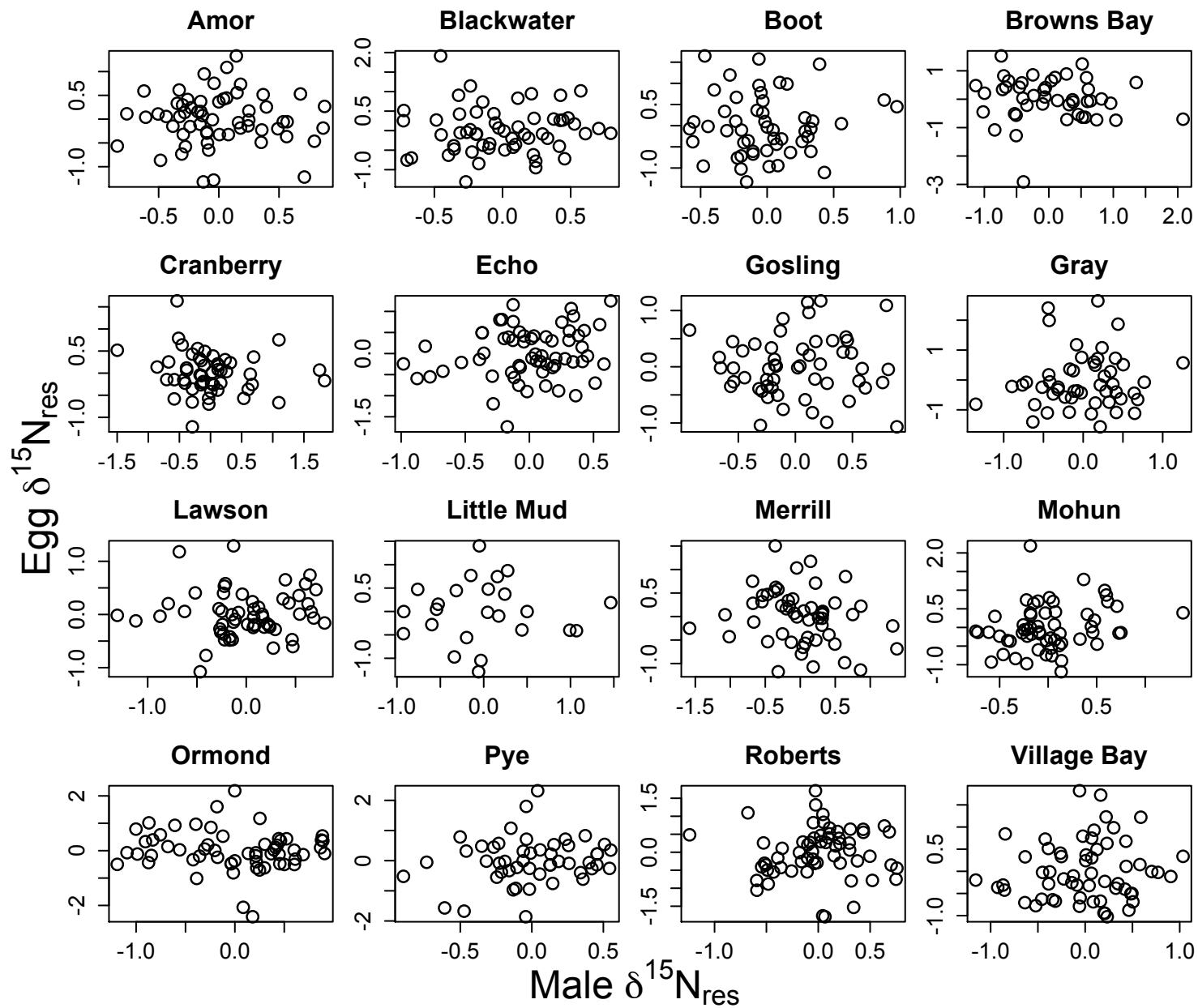

Supplement: Supplementary file 2 — Figure S2. Relationships between male and egg N in each of 16 lakes. [file ece30005-3352-sd2.pdf]
