# Supplementary material for: Widespread positive but weak assortative mating by diet within stickleback populations
Source: Ecol Evol. 2015 Jul 22;5(16):3352–63. doi: 10.1002/ece3.1609 (PMC4569031; doi:10.1002/ece3.1609)
Supplement: Supplementary file 3 — Table S1. Results of linear models using habitat characteristics (nest depth and the presence of vegetation) to predict male and egg C and N. [file ece30005-3352-sd3.pdf]

Supplemental Table 1. Results of linear models using habitat characteristics (nest depth and the presence of vegetation) to predict male and egg δ^{13}C and δ^{15}N .

	Male						Egg					
	δ^{13}C			$\delta^{15}N_{res}$			δ^{13}C			$\delta^{15}N_{res}$		
Lake	Depth	Veg.	R^2	Depth	Veg.	R^2	Depth	Veg.	R^2	Depth	Veg.	R^2
Amor			0.01			0.04	$\Uparrow$	$\uparrow$	0.13			0.00
Blackwater			0.02			0.01			0.00			0.05
Boot			0.05			0.02			0.01			0.00
Brown's Bay			0.04			0.02			0.01	$\Uparrow$		0.09
Cranberry	$\Downarrow$		0.11			0.04			0.02			0.02
Echo			0.00	$\Uparrow$		0.09			0.00			0.03
Gosling			0.01			0.00			0.04			0.02
Gray			0.01			0.03			0.05			0.03
Lawson			0.01			0.00			0.00			0.04
Little Mud			0.01			0.08			0.00			0.012
Merrill			0.02	$\Downarrow$		0.11			0.01		$\Uparrow$	0.12
Mohun	$\uparrow$		0.07			0.01			0.04			0.01
Ormond			0.04			0.03			0.02			0.02
Pye		$\uparrow$	0.07			0.02			0.06			0.07
Roberts			0.00	$\Uparrow$	$\Downarrow$	0.14			0.03			0.5
Village Bay		$\downarrow$	0.06	$\Uparrow$	$\Downarrow$	0.15			0.02			0.02

Arrows indicate positive or negative relationships with between each variable and the isotope ($p < 0.1$: $\uparrow$ or $\downarrow$; $p < 0.05$: $\Uparrow$ or $\Downarrow$). Positive relationships with δ^{13}C imply that the variable is associated with more littoral diets, while positive relationships with $\delta^{15}N_{res}$ indicate that it is associated with higher relative trophic level.
